# Predictors of Recent Alcohol and Substance Use Among Adolescent Girls and Young Women in Namibia

**DOI:** 10.3390/epidemiologia6030034

**Published:** 2025-07-09

**Authors:** Enos Moyo, Hadrian Mangwana, Endalkachew Melese, Simon Takawira, Bernadette Harases, Rosalia Indongo, Perseverance Moyo, Kopano Robert, Tafadzwa Dzinamarira

**Affiliations:** 1 School of Nursing & Public Health, College of Health Sciences, University of Kwa-Zulu Natal, Durban 4001, South Africa; moyoenos@yahoo.co.uk; 2 Project HOPE Namibia, Windhoek 10005, Namibia; 3 Project HOPE—The People-to-People Health Foundation Inc., Windhoek 10005, Namibia; 4 Medical Centre Oshakati, Oshakati 15001, Namibia; moyoperseverance@gmail.com; 5 School of Nursing, Faculty of Health, Humanities, & Social Sciences, Welwitschia University, Windhoek 10005, Namibia; rkpone2000@gmail.com; 6 School of Health Systems and Public Health, Faculty of Health Sciences, University of Pretoria, Pretoria 0028, South Africa; 7 ICAP at Columbia University, Lusaka 10101, Zambia; 8 Africa Centre for Inclusive Health Management, Stellenbosch University, Stellenbosch 7600, South Africa

**Keywords:** adolescent girls and young women, alcohol, substance use, rate, associated factors

## Abstract

**Background:** Adolescent girls and young women (AGYW) who engage in alcohol and substance abuse face more significant health and social consequences compared to the general population. This study evaluated the prevalence and associated factors of alcohol abuse and substance use among AGYW in Namibia. **Methods:** We conducted a retrospective analysis of programmatic data from AGYW aged 10–24 who participated in the Determined, Resilient, Empowered AIDS-free, Mentored, and Safe (DREAMS) component of the Reducing HIV Vulnerability: Integrated Child and Youth Health (REACH) Project HOPE Namibia from March to December 2024. Data analysis was conducted employing chi-squared tests alongside binomial and multinomial logistic regression. **Results:** Among the 19,662 participants included in this analysis, 2068 (10.5%) abused alcohol and/or substances in the previous six months. Participants who were HIV-negative or did not know their status (AOR = 1.57, 95% CI (1.15–2.14), and AOR = 1.50, 95% CI (109–2.07), respectively), from outside Windhoek, those who had failed or repeated school in the previous year (COR = 1.77, 95% CI (1.54–2.05)), those not disabled (AOR = 1.27, 95% CI (1.06–1.52)), those who had dropped out of school or had completed their studies, and those with no adult emotional support (AOR = 1.25, 95% CI (1.11–1.40)), were more likely to have abused alcohol and/or substances recently. In contrast, participants who were not depressed were less likely to have recently abused alcohol and substances. **Conclusions:** The prioritization of strategies to identify AGYW experiencing depression and to provide them with treatment is essential. Moreover, it is important to encourage parents and guardians to provide emotional support to AGYW, as it prevents them from abusing alcohol and substances.

## 1. Introduction

In 2022, there were 292 million drug users worldwide between the ages of 15 and 64 years, representing a 20% rise from the preceding ten years [1]. The majority of individuals undergoing treatment for drug and substance abuse (DSA) disorders in Africa are under the age of 35 [1]. In sub-Saharan Africa, estimates of alcohol use among school-going adolescents range from 10% to 44% [2]. A Namibian study revealed that about 75% of school-going adolescents who used alcohol also used tobacco [3]. Despite a higher prevalence of drug injection among men globally, women who engage in this behavior experience more health and social repercussions [1]. The vulnerability of women arises from traditional gender roles and power structures, which may exacerbate unsafe sexual and injecting behaviors [4]. Women who inject drugs often have male intimate partners who introduce them to substance use and may request these partners to administer injections [1]. Women who use substances, including those who inject, are susceptible to gender-based violence and sexual abuse from intimate partners, other substance users, law enforcement officers, and substance providers [5].

Adolescents exhibit a higher likelihood of illicit substance use than other age groups due to their inclination for experimentation, curiosity, susceptibility to peer pressure, rebellion against authority, and lower self-esteem [6]. The prevalence of DSA usage peaks among individuals aged 18 to 25, with initiation often occurring during adolescence [7]. Studies have identified several risk factors for DSA, including early onset of behavioral and mental health issues, peer pressure, insufficient parental guidance and relationships, dysfunctional family dynamics, and limited opportunities [6,8]. DSA has risen due to the COVID-19 pandemic and the subsequent lockdowns. The COVID-19 pandemic resulted in various socioeconomic challenges, such as job loss, bereavement, and increased indoor confinement, all of which contributed to heightened stress and other mental health issues. The psychological consequences may have increased DSA [9]. Factors such as high self-esteem, religious involvement, self-control, parental supervision, academic proficiency, and anti-drug use regulations protect adolescents and young adults from DSA [10,11,12].

DSA presents multiple negative consequences for adolescents and young adults. DSA impedes the development of critical thinking and essential skills, complicating the transition to adulthood for young individuals engaged in DSA [13]. DSA correlates with school absenteeism, diminished academic performance, and reduced academic self-efficacy [14]. DSA negatively impacts sexual and reproductive health outcomes. Young individuals who engage in substance abuse are more likely to report a lack of condom use, an increased number of sexual partners, higher rates of sexually transmitted infections (STIs), involvement in non-consensual sexual activities, and violence against intimate partners [15]. Furthermore, DSA is associated with adverse reproductive outcomes, such as unintended pregnancies, preterm births, and morbidity and mortality in both mothers and newborns [16].

While numerous studies have explored alcohol and drug use among adolescents and young people in sub-Saharan Africa, there is a paucity of research focusing specifically on adolescent girls and young women (AGYW). Research on the consequences of alcohol and substance use within this population group is essential to guide policymakers in developing effective prevention strategies. This study aimed to assess the prevalence and associated factors of alcohol and/or substance use among AGYW who participated in the Determined, Resilient, Empowered AIDS-free, Mentored, and Safe (DREAMS) component of the Reducing HIV Vulnerability: Integrated Child and Youth Health (REACH) Project Hope Namibia (PHN) activity. REACH PHN was an integrated DREAMS and Orphan and Vulnerable Children (OVC) initiative, funded by the United States of America’s President’s Emergency Plan for AIDS Relief (PEPFAR) through the United States Agency for International Development (USAID) and implemented by a PHN-led consortium. REACH PHN was awarded on 31 July 2023 and was implemented in the Khomas, Ohangwena, Omusati, Oshana, Oshikoto, and Zambezi regions.

## 2. Methods

### 2.1. Study Design

This study is a retrospective cross-sectional secondary analysis of the DREAMS component of REACH PHN’s enrolment and needs assessment data collected between March and December 2024.

#### 2.1.1. Program Intervention and Population

The DREAMS component of REACH PHN aimed to prevent new HIV infections among AGYW and was implemented in Khomas, Oshikoto, Zambezi, and Oshana regions.

Standardized PEPFAR eligibility criteria (see Table 1 below) were used to determine eligibility for the DREAMS program, with interventions guided by the PEPFAR Namibia DREAMS layering table. Alcohol or substance use (ages 10–14) and misuse (ages 15–24) were among the eligibility criteria for the DREAMS component of the REACH PHN.

The DREAMS program addressed factors that increase HIV vulnerability among AGYW, such as gender-based violence, economic exclusion, and limited access to health services. This was achieved by delivering a core package of age-appropriate ‘primary’ interventions for all AGYW, alongside ‘secondary’ interventions tailored to the specific needs of DREAMS-eligible AGYW aged 10 to 24 years. Eligibility for secondary interventions was determined based on the REACH PHN enrolment and needs assessment form, conducted every six months. Additionally, interventions aimed at strengthening families and reducing risks among sexual partners of AGYW were provided. However, education on the dangers of alcohol or substance use and misuse was not a core component of DREAMS interventions, and the school curriculum also does not sufficiently address this issue.

#### 2.1.2. Data Source

Anonymized data were obtained from the REACH PHN DREAMS enrolment and needs assessment program database. Some data collected during the programs included programmatic details, participants’ sociodemographic characteristics, economic factors, and health-related characteristics.

#### 2.1.3. Participant Characteristics

Participants’ characteristics included age group, disability status, district, educational enrolment status, academic progress, survival status of parents, current living arrangement, HIV status, and self-consideration of HIV risk. Furthermore, the other participant variables used in the study were the availability of emotional support from an adult, household hunger score, self-efficacy/resilience/empowerment score, and depression score. The self-efficacy/resilience/empowerment score and the depression score were computed by adding the total scores of the questions for each construct. Self-efficacy/resilience/empowerment scores were then categorized into ‘poor,’ and ‘good,’ while depression scores were categorized into ‘Depressed’ and ‘Not depressed.’

#### 2.1.4. Outcome Variable

The dependent variable in this study was alcohol and/or substance abuse. Recent alcohol and/or substance abuse was defined as abusing these substances in the previous six months. Seven statements were asked to determine whether a participant was abusing alcohol and/or substances. The statements were ‘I felt the need to cut down on my drinking or recreational drug use,’ ‘People annoyed me by criticizing my drinking or recreational drug use,’ ‘I felt bad or guilty about drinking or using the recreational substance,’ ‘I used a drink or recreational drug to steady my nerves or get rid of a hangover,’ ‘I had sex while taking alcohol or recreational drug,’ ‘I forgot the things I have done while using alcohol,’ and ‘I have struggled financially because of alcohol or substance use.’ The answers to these questions were ‘Yes’ or ‘No.’ ‘Yes’ was assigned code ‘2’, whereas ‘No’ was assigned to the code ‘1.’ Answering yes to any of the seven questions was considered to be an alcohol and/or substance abuse problem.

#### 2.1.5. Explanatory Variables

This study used thirteen independent variables covering the participants’ demographics, socioeconomic status, and health-related characteristics. We chose the variables based on their relevance and significance to alcohol and/or substance use among AGYW.

#### 2.1.6. Data Quality Assurance

The digital system facilitated the automatic generation of the Unique Identifier Code (BioID), implemented automated skip rules, and conducted validation checks for variables such as age and sex, and constraints for mandatory questions. The digital system minimized transcription errors, thereby improving data completeness and quality. Data quality assurance (DQA) mechanisms included periodic programmatic spot checks, desk reviews, data quality reviews, and field monitoring by district and regional teams to ensure that reported data met minimum quality standards.

#### 2.1.7. Criteria for Inclusion in Data Analysis

Of the 21,689 DREAMS participants with completed enrolment and needs assessment forms, we excluded those with more than half of their variable responses missing. The remaining 19,662 participants were included in the data analysis.

#### 2.1.8. Data Analysis

Data were exported from the District Health Information System 2 (DHIS2) to IBM Statistical Package for the Social Sciences (SPSS) version 29 for subsequent analysis. Descriptive statistics, including percentages and frequencies, were utilized to analyze nominal and ordinal data. Chi-square tests assessed the relationships between recent alcohol and substance use and the participants’ characteristics. We analyzed characteristics with a *p*-value of less than or equal to 0.05 in chi-square tests via bivariate logistic regression to assess the strength of their associations with recent alcohol abuse and substance use. Characteristics showing statistically significant associations with recent alcohol and substance abuse, as indicated by a *p*-value below 0.05 in binomial logistic regression, were used in multinomial logistic regression to calculate the adjusted odds ratios. While the participant’s academic progress was statistically significant in binomial regression, it was excluded from multinomial regression due to a substantial number of participants not responding. Excluding all participants with no responses would have resulted in a markedly reduced sample size for the multinomial regression analysis.

#### 2.1.9. Ethical Considerations

REACH PHN was approved by the Namibian Ministry of Health and Social Services (MHSS), the Ministry of Education, Arts, and Culture (MoEAC), the Ministry of Gender Equality, Poverty Eradication and Social Welfare (MGEPESW), and the Ministry of Sport, Youth and National Service (MSYNS). Enrolment in the program was entirely voluntary. All minors in the program provided assent, and their parents or caregivers granted consent. AGYW of legal age completed a consent form. Data were only collected from the participants after informed consent was obtained. PHN implements a comprehensive privacy management framework by mandating that all personnel sign a Non-Disclosure Agreement, safeguarding all collected data. Access to DHIS2 was granted based on defined roles and criteria. Each user was assigned a unique username and password-protected login credentials. De-identified or aggregated data were employed when data sharing was necessary. Approval from an institutional review board was not required for the secondary data analysis due to the utilization of anonymous programmatic data.

## 3. Results

### 3.1. Characteristics of Participants

Most of the 20,560 participants included in this analysis were aged 10–14 (*n* = 11,640; 59.2%), not disabled (*n* = 18,022; 91.7%), enrolled at an educational institution (*n* = 17,294; 88.0%), had passed their previous academic year (*n* = 11,811; 60.1%), had one or both parents alive (*n* = 15,084; 76.7%), lived with their parents or caregivers (*n* = 14,882; 75.7%), and had adult emotional support (*n* = 16,080; 81.8%). Furthermore, most participants were from households with little or no hunger (*n* = 13,220; 67.2%), did not know their HIV status (*n* = 12,753; 64.9%), did not consider themselves at risk of HIV (*n* = 17,244; 87.7%), had good self-esteem (*n* = 16,918; 86.0%), and they were not depressed (*n* = 13,802; 70.2%). More details are in Table 2.

### 3.2. Alcohol and/or Substance Use and Abuse Among Participants

Among the 19,662 participants included in this analysis, 2068 (10.5%) participants abused alcohol and/or substances in the previous six months, with a 95% confidence interval (CI) (10.1–10.9%), while 17,594 (89.5%) did not, 95% CI (89.1–89.9%). A total of 8950 (45.5%) reported using alcohol and/or substances in the previous six months. More details are in Table 3.

#### 3.2.1. Frequency Distribution of Alcohol and/or Substance Abuse by District Among Participants

Onandjokwe had the highest proportion of participants who abused alcohol or substances during the previous six months (*n* = 546; 17.8%), while Windhoek had the lowest (*n* = 371; 4.9%). More details are in Table 4.

#### 3.2.2. Factors Associated with Alcohol and/or Substance Abuse Among Participants

Chi-square tests revealed statistically significant associations between recent alcohol and/or substance abuse and the participant’s age group, district, educational institution enrolment status, previous year academic progress, current living arrangement, HIV status, self-consideration of HIV risk, household hunger score, disability, adult emotional support, and depression (*p* < 0.05). However, no associations were noted between recent alcohol and/or substance abuse and participants’ self-efficacy/resilience/empowerment score (*p* > 0.05). HIV-negative participants and those who did not know their HIV status had a higher likelihood of reporting recent alcohol and substance abuse than those who were HIV-positive, AOR = 1.57, 95% CI (1.15–2.14), and AOR = 1.50, 95% CI (109–2.07), respectively. Non-disabled participants and those without adult emotional support were more likely to report recent alcohol and/or substance abuse, AOR = 1.27, 95% CI (1.06–1.52), and AOR = 1.25, 95% CI (1.11–1.40), respectively. Furthermore, participants from outside Windhoek had a higher likelihood of recent alcohol and/or substance abuse than those from Windhoek. Although an association was noted between school enrolment and recent alcohol and substance abuse in bivariate logistic regression, the associations were not significant in the adjusted analysis. In contrast, participants aged 10–14, those from households with little or no hunger, and those not depressed were less likely to have recently abused alcohol and/or substances, AOR = 0.46, 95% CI (0.38–0.56), AOR = 0.75, 95% CI (0.63–0.91), and AOR = 0.67, 95% CI (0.60–0.73), respectively. More details are in Table 5.

## 4. Discussion

This study revealed that 10.5% of the participants had abused alcohol and/or substances in the previous six months. Omuthiya had the highest alcohol and substance use rate, while Windhoek had the lowest. Participants who were HIV-negative or did not know their status, those from outside Windhoek, those who had failed or repeated in the previous year, those not disabled, those who had dropped out of school or had completed their studies, and those with no adult emotional support were more likely to have abused alcohol and/or substances recently. In contrast, participants aged 10–14, from households with little or no hunger, and those not depressed were less likely to have recently abused alcohol and/or substances.

The rate of 10.5% revealed in this study is lower than the 30.4% reported among adolescent girls in a Ugandan study [17]. The low rate reported in the current study may be attributable to the differences in the study participants and their socioeconomic circumstances. The study revealed that participants from outside Windhoek were more likely to have recently abused alcohol and/or substances. This is possibly a result of the towns being affected more economically due to COVID-19 because of the closure of companies than Windhoek. Most companies might have preferred to keep the branches in Windhoek open since there might be more business there. The economic consequences might have brought depression among AGYW, who might have struggled to obtain money for their education, leading to alcohol and/or substance use. The higher likelihood in the other towns may emanate from variations in the risk and protective factors in different regions. The implementation of laws governing alcohol and substance use among underage youth may be inadequate in certain areas, resulting in increased access and availability of these substances to AGYW.

As expected, participants who had failed or repeated at school the previous year were more likely to have recently abused alcohol and/or substances. This finding concurs with that of an Ethiopian study, which revealed that poor school performance was associated with substance use among high school adolescents [18]. This is because alcohol and substances are known to affect academic performance and self-efficacy [14]. This study revealed that HIV-negative participants had a higher likelihood of recent alcohol and substance abuse. A study conducted in Kenya found that youth living with HIV had a lower prevalence of substance use than their HIV-negative counterparts [19]. This may be due to HIV-positive participants receiving education regarding the side effects of combining antiretroviral therapy with alcohol and/or substances at healthcare facilities during medication collection. This information may have encouraged the avoidance of alcohol and substances [20].

Participants who had dropped out of school and those who had completed their studies were more likely to abuse alcohol and/or substances. This may result from experimentation among the AGYW, who may have nothing to do. Younger adolescents out of school are more likely to explore their identity and sense of self, potentially confusing their identity [21]. Identity confusion may result in risky behaviors, often without a comprehensive evaluation of the potential consequences of such actions [22]. Participants from households with little or no hunger were less likely to have recently abused alcohol and/or substances. This finding contrasts with that of a South African study of women living with HIV, which indicated that food insecurity was associated with a lower likelihood of alcohol and/or substance use [23]. The findings are attributable to depression related to food insecurity, which may increase alcohol and/or substance abuse. The current study revealed that participants without adult emotional support were more likely to report alcohol and/or substance abuse. A study conducted in sub-Saharan Africa revealed that good family communication and good parent–child relationships are required to prevent them from abusing alcohol and/or substances [24].

In this study, participants who were not depressed were less likely to report recent alcohol and/or substance use. This finding is similar to that of a South African study, which revealed that female adolescents who had no depressive symptoms were less likely to use substances [25]. Depression can play a role in both the onset and persistence of alcohol and/or substance use as a coping mechanism for life challenges [26]. Prioritizing strategies to identify AGYW experiencing depression and providing them with treatment is essential for mitigating alcohol and/or substance abuse in this population.

This study has a limitation in that the findings may have been influenced by social desirability bias resulting from the self-reported nature of the responses. A qualitative future study should investigate the underlying factors contributing to alcohol and/or substance abuse among AGYW to improve comprehension of the issue and guide prevention strategies. The cross-sectional design of the study precludes the inference of causal relationships. Considering the large sample size, we believe that the results are generalizable to all AGYW at risk of HIV in Namibia.

## 5. Conclusions

This study revealed that 10.5% of the participants had abused alcohol and/or substances in the previous six months. Participants who were HIV-negative or did not know their status, those who had dropped out of school or completed their studies, those who were not disabled, from outside Windhoek, those who had failed or repeated in the previous year, and those without adult emotional support were more likely to have used alcohol and/or substances recently. The implementation of laws governing alcohol and substance use among underage youth should be promoted. Prioritization of strategies to identify AGYW experiencing depression and to provide them with treatment is essential. Moreover, it is important to encourage parents and guardians to provide emotional support to AGYW, as it prevents them from abusing alcohol and substances.

## Figures and Tables

**Table 1:** DREAMS eligibility criteria *.

10–14 Years	15–19 Years	20–24 Years
▪ Ever had sex. ▪ History of pregnancy. ▪ Experience of sexual violence (lifetime). ▪ Experience of physical or emotional violence (within the last year). ▪ Any alcohol or other substance use. ▪ Out of school. ▪ Orphanhood.	▪ Multiple sexual partners. ▪ History of pregnancy. ▪ STI (diagnosed or treated) ▪ No or irregular condom use. ▪ Transactional sex. ▪ Experience of sexual violence (lifetime). ▪ Alcohol or other substance misuse. ▪ Out of school. ▪ Orphanhood.	▪ Multiple sexual partners. ▪ STI (diagnosed or treated). ▪ No or irregular condom use. ▪ Transactional sex. ▪ Experience of sexual violence (lifetime). ▪ Alcohol or other substance misuse.

* AGYW meeting any one of the age band-specific criteria are eligible for enrolment into the DREAMS component of REACH PHN.

**Table 2:** Characteristics of participants.

Characteristics	Frequency *n* (%)
Participant’s age group (years)	
10–14	11,640 (59.2)
15–19	5261 (26.8)
20–24	2761 (14.0)
District	
Onandjokwe	3066 (15.6)
Tsumeb	777 (4.0)
Oshakati	3087 (15.7)
Katima Mulilo	4012 (20.4)
Omuthiya	1075 (5.5)
Windhoek	7645 (38.9)
Disability	
No	18,022 (91.7)
Yes	1640 (8.3)
School enrolment	
Dropped out	1624 (8.3)
Never been enrolled	49 (0.2)
Completed high school, vocational training, college, or university	695 (3.5)
Yes, enrolled	17,294 (88.0)
Academic progress	
Failed/repeated	1858 (9.4)
Do not know	22 (0.1)
Passed	11,811 (60.1)
Refused to answer/missing information	5971 (30.4)
Living status of biological parents	
Both dead	190 (1.0)
One or both alive	15,084 (76.7)
Refused to answer/missing information	4388 (22.3)
Living arrangement	
Lives away from parents/caregivers	346 (1.8)
Lives in a child-headed household	32 (0.2)
Lives with parents/caregivers	14,882 (75.7)
Refused to answer/missing information	4402 (22.4)
Household hunger score	
Little or no hunger	13,220 (67.2)
Moderate hunger	5231 (26.6)
Severe hunger	1211 (6.2)
HIV status	
Negative	6476 (32.9)
Don’t know	12,753 (64.9)
Positive	433 (2.2)
Do you consider yourself at risk of HIV?	
Yes	1823 (9.3)
No	17,244 (87.7)
Refused to answer/missing information	544 (2.8)
Self-Esteem/Resilience/Empowerment	
Poor	2744 (14.0)
Good	16,918 (86.0)
Depressed	
No	13,802 (70.2)
Yes	5836 (29.7)
Refused to answer/missing information	24 (0.1)
Adult emotional support	
Absent	3582 (18.2)
Present	16,080 (81.8)

**Table 3:** Frequency distribution of alcohol and substance use and abuse among participants.

Substance	Frequency	
	No *n* (%)	Yes *n* (%)
Alcohol and/or drug use	10,712 (54.5)	8950 (45.5)
Alcohol use	10,785 (54.9)	8877 (45.1)
Other recreational drug use	19,488 (99.1)	174 (0.9)
Alcohol and/or drug misuse	17,594 (89.5)	2068 (10.5)

**Table 4:** Frequency distribution of alcohol and/or substance abuse by district among participants.

District	Alcohol and Substance Use	
	No *n* (%)	Yes *n* (%)
Onandjokwe	2520 (82.2)	546 (17.8)
Tsumeb	662 (85.2)	115 (14.8)
Oshakati	2663 (86.3)	424 (13.7)
Katima Mulilo	3481 (86.8)	531 (13.2)
Omuthiya	994 (92.5)	81 (7.5)
Windhoek	7274 (95.1)	371 (4.9)
TOTAL	17,594 (89.5)	2068 (10.5)

**Table 5:** Factors associated with alcohol and/or substance abuse among participants.

	Crude Odds Ratios	95% CI *	Adjusted ** Odds Ratios	95% CI *	Chi-Square Test *p*-Value
Participant’s age group (years)					**<0.01**
10–14	**0.36**	**0.32–0.41**	**0.46**	**0.38–0.56**	
15–19	0.95	0.84–1.08	1.01	0.85–1.19	
20–24	Reference	Reference	Reference	Reference	
District					**<0.01**
Onandjokwe	**4.25**	**3.70–4.88**	**4.29**	**3.70–4.97**	
Tsumeb	**3.41**	**2.72–4.26**	**2.74**	**2.18–3.45**	
Oshakati	**3.12**	**2.70–3.61**	**2.97**	**2.55–3.45**	
Katima Mulilo	**2.99**	**2.60–3.44**	**2.34**	**2.02–2.70**	
Omuthiya	**1.60**	**1.25–2.05**	**1.46**	**1.13–1.89**	
Windhoek	Reference	Reference	Reference	Reference	
Disability					**0.013**
No	**1.25**	**1.05–1.50**	**1.27**	**1.06–1.52**	
Yes	Reference	Reference	Reference	Reference	
School enrolment					**<0.01**
Dropped out	**2.30**	**2.01–2.62**	1.17	0.98–1.40	
Never been enrolled	1.32	0.56–3.10	0.69	0.29–1.65	
Completed high school, vocational training, college, or university	**1.35**	**1.07–1.70**	0.78	0.60–1.02	
Yes, enrolled	Reference	Reference	Reference	Reference	
Academic progress					**<0.01**
Failed/repeated	**1.77**	**1.54–2.05**	NI	NI	
Do not know	1.05	0.25–4.49	NI	NI	
Passed	Reference	Reference	Reference	Reference	
Living status of biological parents					**0.05**
Both dead	1.53	0.99–2.35	NI	NI	
One or both alive	Reference	Reference	NI	NI	
Living arrangement					**<0.01**
Lives away from parents/caregivers	0.74	0.49–1.14	NI	NI	
Lives in a child-headed household	1.94	0.74–5.03	NI	NI	
Lives with parents/caregivers	Reference	Reference	Reference	Reference	
Household hunger score					**<0.01**
Little or no hunger	**0.72**	**0.60–0.86**	**0.75**	**0.63–0.91**	
Moderate hunger	0.86	0.71–1.04	0.92	0.76–1.12	
Severe hunger	Reference	Reference	Reference	Reference	
HIV status					**<0.01**
Negative	**1.38**	**1.02–1.87**	**1.57**	**1.15–2.14**	
Don’t know	**0.67**	**0.50–0.91**	**1.50**	**1.09–2.07**	
Positive	Reference	Reference	Reference	Reference	
Self-Esteem/Resilience/Empowerment					0.76
Poor	NC	NC	NI	NI	
Moderate	NC	NC	NI	NI	
Good	NC	NC	NI	NI	
Depressed					**<0.01**
No	**0.66**	**0.60–0.73**	**0.67**	**0.60–0.73**	
Yes	Reference	Reference	Reference	Reference	
Adult emotional support					**<0.01**
Absent	**1.34**	**1.20–1.50**	**1.25**	**1.11–1.40**	
Present	Reference	Reference	Reference	Reference	

NC—not computed; NI—not included; * CI is the 95% confidence interval; ** adjusted for the participant’s district, age group, school enrolment, HIV status, disability, household hunger score, adult emotional support, and depression. Bold numbers—Statistically significant results

## Data Availability

Data used for the study can be obtained on reasonable request from the lead author.
